# Prospective longitudinal outcomes of quality of life after laparoscopic radical prostatectomy compared with retropubic radical prostatectomy

**DOI:** 10.1186/s12955-017-0835-1

**Published:** 2018-01-05

**Authors:** Katsuyoshi Hashine, Toshio Kakuda, Shunsuke Iuchi, Tadanori Hosokawa, Iku Ninomiya

**Affiliations:** Department of Urology, National Hospital Organization Shikoku Cancer Center, 160 Minamiumemoto, Matsuyama, 791-0280 Japan

**Keywords:** Health-related quality of life, Laparoscopic radical prostatectomy, Urinary function, Urinary bother

## Abstract

**Background:**

There have been few reports on health-related quality of life (HRQOL) after laparoscopic radical prostatectomy (LRP) in Japanese patients. The aim of this study is to assess changes in HRQOL during 36 months after LRP compared with retropubic radical prostatectomy (RRP).

**Methods:**

The subjects were 105 consecutive patients treated with LRP between 2011 and 2012. HRQOL was evaluated using the International Prostate Symptom Score (IPSS), Medical Outcome Study 8-Items Short Form Health Survey (SF-8), and Expanded Prostate Cancer Index Composite (EPIC) at baseline and 1, 3, 6, 12 and 36 months after surgery. These results were compared with data for 107 consecutive patients treated with RRP between 2005 and 2007. The comparison between LRP and RRP was examined at every time point by Mann-Whitney U-test and chi-square test. Multiple linear regression analysis was used to identify independent factors related to the urinary domain in EPIC.

**Results:**

The IPSS change was similar in both groups. The LRP group had a better SF-8 mental component summary score at baseline and a better SF-8 physical component summary score at 1 month after surgery. In EPIC, urinary function and bother were worse after LRP, but improved at 12 months and did not differ significantly from those after RRP; however, these factors then worsened again at 36 months after LRP. Urinary incontinence was also worse at 36 months after LRP, compared to RRP. In patients treated with nerve-sparing surgery, urinary function and urinary incontinence were similar and good at 12 and 36 months in both groups. Bowel function and bother, and sexual function and bother were similar in both groups and showed no changes from 12 to 36 months. Age and salvage radiotherapy were independent predictors of incontinence (daily use of two or more pads) in multivariate analysis. Surgical procedure was not an independent factor for incontinence, but incontinence defined as use of one pad or more was associated with the surgical procedure.

**Conclusions:**

Urinary function and bother at 36 months were worse after LRP than after RRP. Age, salvage radiotherapy and surgical procedure were associated with urinary incontinence after 36 months.

## Background

Prostate cancer is increasing in Japan and the prevalence in 2016 may be the highest among malignancies in males. Surgery and radiotherapy are used for non-metastatic prostate cancer, with retropubic radical prostatectomy (RRP) originally being the common surgery. More recently, laparoscopic radical prostatectomy (LRP) has been introduced in Japan [1], and there has been a gradual increase in use of LRP and an improvement in techniques. In our hospital, we began to use LRP in 2009 and 352 patients underwent this procedure up to 2014.

Evaluation of surgical success should include assessment of both oncological and functional outcomes. Oncological outcomes of LRP and RRP have been widely examined [2–5], but there are fewer reports on functional outcomes [6–8]. Functional outcomes after LRP have been shown to be similar or worse than those after RRP [9, 10]. Namiki et al. found delayed recoveries of urinary function and sexual function in LRP compared to RRP [7], whereas Ball et al. reported similar outcomes for urinary incontinence after LRP and RRP [9]. In an earlier study, we investigated postoperative health-related quality of life (HRQOL) to evaluate functional outcomes in the first year after LRP [10]. The results showed poor urinary function, and especially urinary incontinence, after LRP. In the current study, we hypothesized that QOL at 3 years after LRP would recover to baseline, and we examined the factors associated with QOL after LRP and compared the results to similar data after RRP.

## Methods

LRP has been performed for 352 patients with clinically localized prostate cancer at our hospital since May 2009. This study started with the 78th patient in this period and was planned for 100 cases. Ultimately, 105 consecutive patients were treated between January 2011 and June 2012. The indication for surgery and the surgical technique have been described previously [10]. During this period, only LRP was performed because RRP had been shifted completely to LRP. For this reason, comparative data were used for 107 consecutive patients treated with RRP between October 2005 and July 2007. In this period, only RRP was performed, and the first patient treated in the historical cohort was the 270th patient treated with RRP.

A HRQOL survey was performed before surgery (baseline = 0) and at 1, 3, 6, 12 and 36 months after surgery. The survey was performed as a self-assessment with no interview at the time of visiting hospital. If the patient was observed at another clinic, we mailed the QOL questionnaire to obtain the answers. The Japanese version of the Medical Outcome Study 8-Items Short Form Health Survey (SF-8) was used to assess general HRQOL. The SF-8 consists of eight scales and generates two summary measures: a physical component summary (PCS) and a mental component summary (MCS). These two summary scores were used for comparison between LRP and RRP. The International Prostate Symptom Score (IPSS) and the Expanded Prostate Cancer Index Composite (EPIC) were used for assessment of disease-specific HRQOL. The IPSS consists of 7 items for filling and voiding symptoms. EPIC is a 50-item questionnaire that quantifies prostate cancer-specific HRQOL in 8 separate domains. In this study, the domains of hormone function and bother were omitted because there were few patients who underwent androgen deprivation therapy (ADT), which was used in a neoadjuvant setting only. All scales of the SF-8 are shown by comparison with the average in the general Japanese population. PCS and MCS scores were calculated using the associated scoring program. Each item in the IPSS is classified in six phases from 0 to 5 and IPSS is expressed on a scale of 0 (excellent) to 35 (terrible). All EPIC scores were linearly transformed to a scale of 0 (lowest) to 100 (highest). The HRQOL survey was returned by 83.8%, 82.9%, 85.7%, 87.6%, 85.7% and 89.5% patients at 0, 1, 3, 6, 12 and 36 months after surgery.

Urinary incontinence was evaluated based on daily use of pads, in addition to the EPIC urinary incontinence score (incontinence-EPIC), and defined as use of ≥2 pads each day (incontinence-pad). In the study periods, PSA failure (PSA >0.2 ng/ml) occurred in 16 patients after LRP, of whom 7 were treated by salvage radiotherapy and 5 by ADT; and in 31 patients after RRP, of whom 4 and 21 were treated by the respective therapies.

The comparison between LRP and RRP was examined at every point. Changes in each group are shown as the difference from baseline at each time point. Group comparisons were performed by Mann-Whitney U-test and chi-square test, with two-tailed *P*-values <0.05 considered significant. Multiple linear regression analysis was used to identify independent factors related to the urinary domain in EPIC. In multivariate analysis, age and Gleason score were used as continuous parameters, and surgical procedure (LRP vs. RRP), resection margin (positive vs. negative), nerve sparing surgery (with vs. without), pathological stage (pT2 vs. pT3), salvage radiotherapy (yes vs. no), IPSS (low vs. high), PSA failure (yes vs. no), and ADT (yes vs. no) were used as categorical parameters. Using incontinence-EPIC and -pad, a multiple logistic regression analysis was performed with the same parameters. SPSS ver. 20.0 J was used for all statistical analyses. This survey was approved by the Institutional Review Board and written informed consent was obtained from all patients.

## Results

The characteristics of the patients are shown in Table 1. There were no differences in age, Gleason Score, use of neoadjuvant ADT, nerve-sparing surgery, salvage radiotherapy (RT) and positive resection margin, but PSA and clinical stage were worse and use of adjuvant ADT was more common in the RRP group. The results for the first year after surgery have been described elsewhere [7], and thus are only briefly explained here. The IPSS change was similar in both groups. The LRP group had a better baseline SF-8 MCS score and a better SF-8 PCS score at 1 month after surgery. In EPIC, obstructive/irritative symptoms did not differ between LRP and RRP, but urinary incontinence-EPIC after LRP was worse until 12 months after surgery. Urinary bother was worse in the LRP group at 1 and 3 months, but did not differ among the groups thereafter.

**Table 1:** Characteristics of patients undergoing laparoscopic radical prostatectomy (LRP) and retropubic radical prostatectomy (RRP)

	LRP	RRP	*P* value
Number	105	107	
Age (years)			0.255
Median (range)	66.0 (51–78)	67.0 (51–79)	
Clinical stage			<0.001
T1	74	65	
T2	31	34	
T3	0	8	
PSA (ng/ml)			0.007
Median	7.56 (1.66–28.40)	9.77 (1.29–88.68)	
Gleason score (biopsy)			0.111
≤ 6	30	19	
7	43	38	
≥ 8	32	50	
NAADT	10	6	0.310
Nerve sparing	12	12	1.000
PRM	30	38	0.305
PSA failure	16	31	0.073
Salvage RT	7	4	0.274
AADT	5	21	0.009

*NAADT* neoadjuvant androgen deprivation therapy, *PRM* positive resection margin, *RT* radiotherapy, *AADT* adjuvant androgen deprivation therapy

**Fig. 1:**
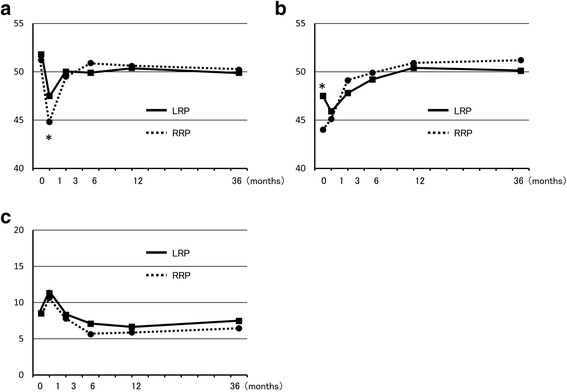
Dependence of (**a**) physical component summary score, (**b**) mental component summary score, and (**c**) International Prostate Symptom Score on the surgical method. *P < 0.05 for LRP vs. RRP

**Fig. 2:**
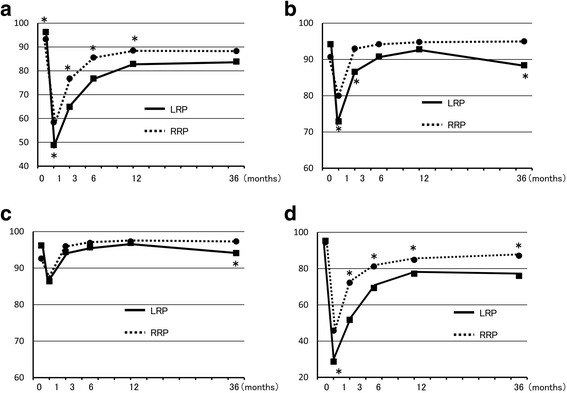
Dependence of EPIC scores for (**a**) urinary function, (**b**) urinary bother, (**c**) urinary irritative/obstructive symptoms, and (**d**) urinary incontinence on the surgical method. *P < 0.05 for LRP vs. RRP

**Fig. 3:**
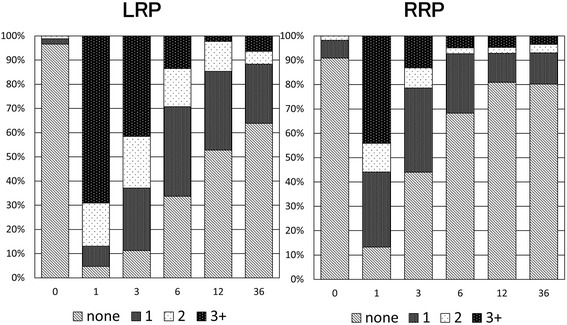
Dependence of use of a pad on the surgical method. None: no pad use, 3+: use of ≥3 pads each day

**Fig. 4:**
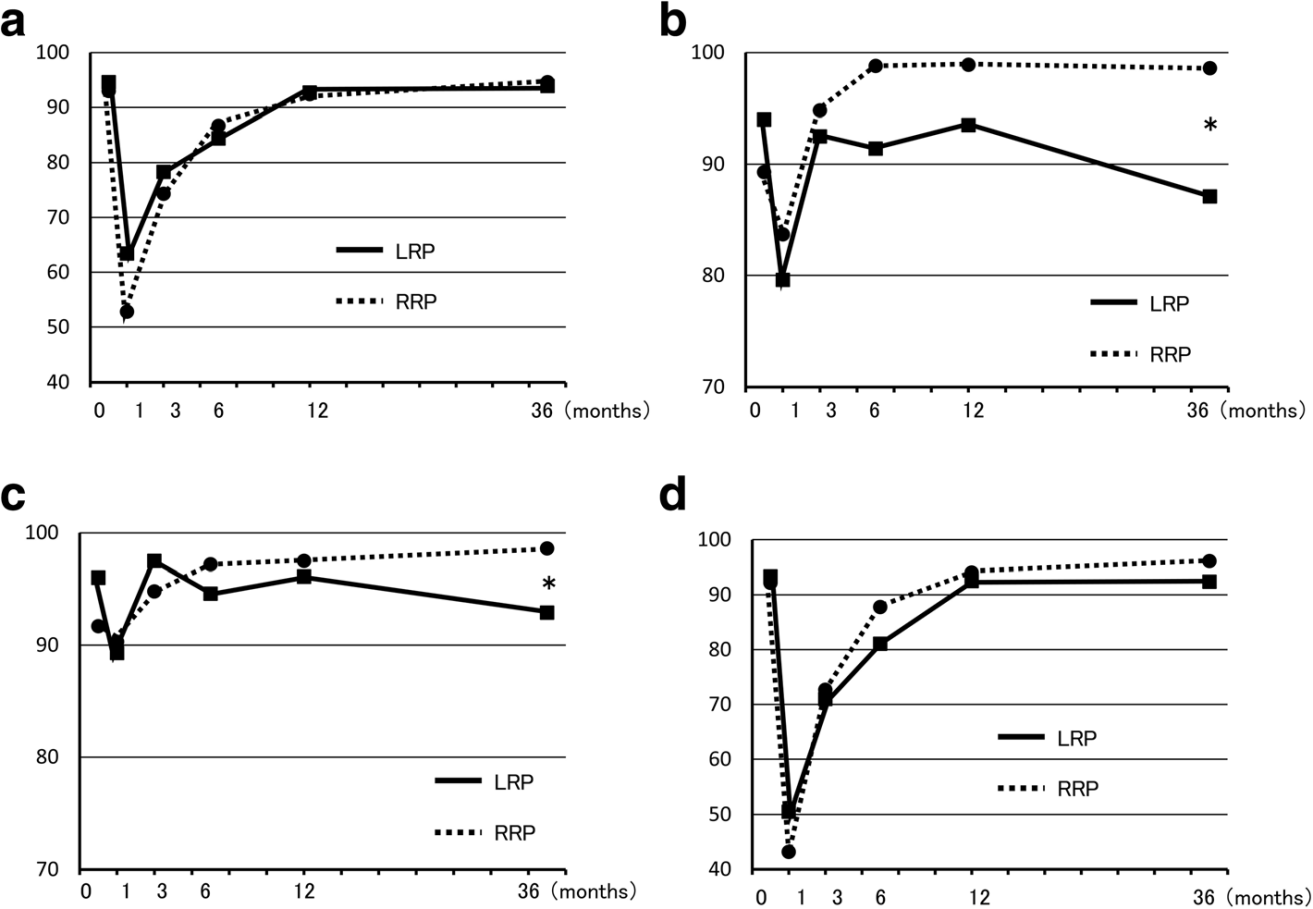
Dependence of EPIC scores for (**a**) urinary function, (**b**) urinary bother, (**c**) urinary irritative/obstructive symptoms, and (**d**) urinary incontinence on the surgical method in cases treated with nerve-sparing surgery. *P < 0.05 for LRP vs. RRP

**Fig. 5:**
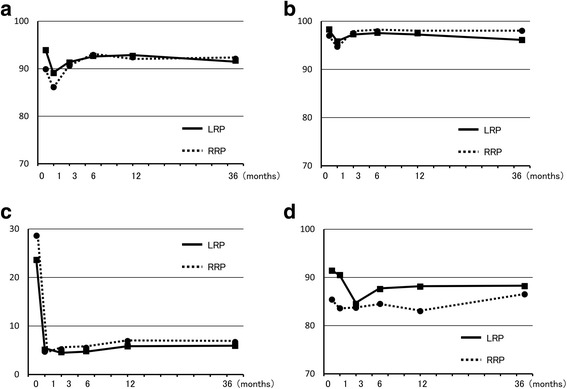
Dependence of EPIC scores for (**a**) bowel function, (**b**) bowel bother, (**c**) sexual function, and (**d**) sexual bother on the surgical method

After 36 months, PCS and MCS in SF-8 were the same after LRP and RRP. IPSS at 36 months was also the same in both groups and showed no change from 12 months (Fig. 1). In EPIC, urinary function after LRP was worse than after RRP, but there was no significant difference at 36 months. In contrast, urinary bother, irritative/obstructive symptoms, and urinary incontinence-EPIC at 36 months were significantly worse after LRP than after RRP, and these parameters were worse at 36 months compared to 12 months after LRP (Fig. 2). Urinary incontinence-pad was similar in both groups, but there were fewer patients with no pad use after RRP (Fig. 3). In patients treated with nerve-sparing surgery, urinary function and urinary incontinence-EPIC were similar and good at 12 and 36 months in both groups, but urinary bother and irritative/obstructive symptoms after 36 months were worse in the LRP group (Fig. 4). Bowel function and bother, and sexual function and bother were similar in both groups and showed no changes from 12 to 36 months (Fig. 5).

In multivariate analysis, urinary incontinence-pad at 36 months was associated with age and salvage RT, but surgical procedure was not an independent factor. If incontinence-pad was defined as daily use of one or more pads, it was associated with age, surgical procedure and salvage RT (Table 2). Surgical procedure was associated with almost all urinary domains at 36 months and was related to negative outcomes. After 1 month, a nerve-sparing procedure was associated with urinary incontinence-EPIC and with a positive outcome. IPSS at baseline was associated with urinary bother and irritative/obstructive symptoms after 3 months and a high score was associated with a negative outcome. Age was a significant factor at all time points and older age was associated with a negative outcome. A negative resection margin and salvage RT were independent factors associated with a negative outcome (Table 3). Among patients without PSA failure, the significant difference in urinary incontinence-EPIC remained between LRP and RRP. However, the difference in scores became small using data for all patients.

**Table 2:** Independent factors associated with urinary incontinence at 36 months by multivariate analysis

Daily use of two pads or more (incontinence-pad)		
	HR	*P* value
Salvage RT (no vs. yes)	10.526 (1.560–71.429)	0.016
Age (continuous)	1.168 (1.006–1.356)	0.041
Daily use of one pad or more		
	HR	*P* value
Age (continuous)	1.141 (1.063–1.225)	<0.001
Surgical procedure (RRP vs. LRP)	2.427 (1.174–5.025)	0.017
Salvage RT (no vs. yes)	3.891 (0.962–15.625)	0.057

*RT* radiotherapy, *RRP* retropubic radical prostatectomy, *LRP* laparoscopic radical prostatectomy

**Table 3:** Independent factors associated with urinary domains in EPIC in multivariate analysis

	Urinary domain			
Factor	Function β, γ	Bother β, γ	Irritative/obstructive β, γ	Incontinence β, γ
At 1 month				
Surgical procedure (ref: RRP)	−0.22*, −0.22*	−0.22**, −0.22*	–	−0.30*, −0.29*
NS (ref: without)	–	–	–	0.17**, 0.17**
At 3 months				
Surgical procedure (ref: RRP)	−0.29*, −0.30*	−0.28*, −0.27*	–	−0.36*, −0.35*
Age (continuous)	−0.22*, −0.20*	–	–	−0.21*, −0.20*
IPSS (ref: low)	–	−0.27*, −0.26*	−0.26*, −0.26*	–
RM (ref: positive)	−0.15**, −0.20*	–	–	–
At 6 months				
Surgical procedure (ref: RRP)	−0.23*, −0.23*	−0.17**, −0.17**	–	−0.23*, −0.13*
Age (continuous)	−0.20*, −0.18*	–	–	−0.23*, −0.23*
RM (ref: positive)	−0.17**, −0.18**	−0.18**, −0.21*	−0.22*, −0.24*	−0.17**, −0.17**
IPSS (ref: low)	–	−0.25*, −0.27*	−0.21*, −0.23*	–
At 12 months				
Surgical procedure (ref: RRP)	−0.19**, −0.18*	–	–	−0.17**, −0.17**
Age (continuous)	−0.17**, −0.19*	–	–	−0.20**, −0.23*
RM (ref: positive)	−0.17**, −0.19*	−0.16**, −0.20*	–	−0.18**, −0.21*
IPSS (ref: low)	−0.16**, −0.19*	−0.21*, −0.22*	−0.21*, −0.20*	−0.15**, −0.20*
SRT (ref: no)	–	−0.18**, −0.17**	−0.18**, −0.17**	–
At 36 months				
Surgical procedure (ref: RRP)	–	−0.30*, −0.28*	−0.24*, −0.22*	−0.21*, −0.23*
Age (continuous)	−0.28*, −0.26*	–	–	−0.24*, −0.22*
SRT (ref: no)	−0.16**, −0.12**	–	–	−0.18**, −0.17**
IPSS (ref: low)	–	−0.29*, −0.27*	−0.30*, −0.29*	–

*NS* nerve sparing procedure, *IPSS* International Prostate Symptom Score, *RM* resection margin, *SRT* salvage radiotherapy

*: *p* < 0.01, **: *p* < 0.05, β; standardized partial regression coefficient, γ: correlation coefficient

## Discussion

The functional outcome after radical prostatectomy is of similar importance to the oncological outcome. In a previous study of functional outcomes until 12 months after surgery, we found that urinary function and bother were worse after LRP compared to RRP, except for patients who underwent nerve-sparing surgery [10]. Based on these results, we concluded that LRP may have a worse influence on urinary function due to limited 2-D vision and limited movement of the forceps through the port, despite the magnified view. The rates of use of a pad in the first year have been reported to be 17% after LRP and 12% after RRP [6], and we obtained similar results. A study using the University of California, Los Angeles, Prostate Cancer Index (UCLA-PCI) showed that urinary function and bother were worse after LRP than after RRP for 1 month, but similar after 3 months [7]. A recent meta-analysis found a rate of 82–100% for urinary continence defined as daily use of 0–1 pads [8].

There are many benefits of LRP, including more accurate dissection due to the magnified view and a clear view due to less bleeding, compared with RRP. Therefore, we predicted that the poorer functional outcomes at 12 months would not be present at 36 months after surgery. However, in the current study, some outcomes at 36 months were worse after LRP compared with RRP, and worse than those at 12 months. Urinary incontinence-EPIC and irritative/obstructive symptoms were significantly worse at 36 months after LRP, but urinary incontinence-pad was the same in both groups at 36 months and did not differ between 12 and 36 months. Among the urinary domains in EPIC, only dripping or leaking urine and waking up to urinate were significant. In multivariate analysis, LRP was a significant factor for worse urinary function and bother based on EPIC scores in almost all periods. Only age and salvage RT were significant factors related to urinary incontinence-pad. However, the surgical procedure became a significant factor when urinary incontinence-pad was defined as use of one pad or more.

The poorer functional outcomes of LRP may be due to a difference in the experience of surgeons performing the surgery. LRP is a more complex procedure than RRP and requires a long learning curve. That is, the learning curve was different in the two groups, since the survey was started after treatment of 78 cases in the LRP group, but after 270 cases in the RRP group. Some investigators have reported that LRP has a worse outcome for urinary incontinence and concluded that urinary incontinence was dependent on surgical technique [11, 12]. There was less experience with LRP in this study, but because the surgical method completely shifted from RRP to LRP, understanding of the anatomy was more advantageous in LRP. The positive resection margin and PSA failure were similar in the two groups and the learning curve does not seem to have affected the results based on these oncological outcomes. However, LRP requires more surgical experience and the learning curve is important.

Another reason for the poorer functional outcomes may be use of more salvage RT after LRP. Salvage RT is a useful treatment for PSA failure after surgery, but in multivariate analysis salvage RT was an independent factor reducing QOL after therapy. Bolla et al. found that adverse effects were more frequent after salvage RT compared to a wait-and see approach [13], and salvage RT seems to have worsened outcomes in the current study. The poorer urinary function after LRP may also be related to general worsening of this function with age. A study of long-term QOL in Japan using UCLA-PCI showed that urinary function and IPSS decreased over 5 years, although the change was not significant [14]. In addition, urinary function significantly decreased after the second year in an analysis of 1788 RRP cases [15]. Similar findings have been obtained at 15 years after surgery [16]. Naselli et al. found that 11% of patients with complete continence after 2 years later developed urinary incontinence [17]. In the current study, age was an independent factor related to urinary function and bother, and the worsening of these functions in the third year after surgery is similar to that observed in the reports above. Generally, QOL worsens with aging, but the reason for the difference at 36 months between LRP and RRP is unclear. However, our results show that the surgical procedure affected outcomes at both 12 and 36 months after surgery.

Comparisons of LRP with robot-assisted radical prostatectomy (RARP) have shown that the procedures have similar oncological outcomes [18, 19], but recent studies suggest that functional outcomes such as incontinence and sexual function are better after RARP [20, 21]. In two randomized controlled trials (RCTs) comparing postoperative QOL for LRP and RARP, Asimakopouloa et al. found that urinary incontinence did not differ significantly [22], but Piglia et al. found significantly better recovery of urinary incontinence and potency after RARP [23]. Despite the difference in conclusions between these two RCTs, most reports find better early recovery of urinary incontinence after RARP.

There were some limitations in the current study. First, the study was prospective, but performed with comparison of patients in different periods. Therefore, the background differed between the two groups. We used multivariate analysis to eliminate background factors as much as possible. More RRP patients were treated with adjuvant ADT, but ADT was not a significant factor in multivariate analysis. A second limitation is that the study was performed in a small cohort. Comparisons between two groups should be analyzed by a matching technique, but we were unable to compare LRP and RRP by matched pair analysis due to the small number of cases after matching, and subsequent lack of statistical significance. For this reason, all data were examined. An alternative was to use data only for patients who provided answers at all time points, but this method also decreased the number of cases for analysis, despite the response rate being over 80% at each time point. In addition, for the urinary domain, independent factors were examined by multivariate analysis, and the influence of background factors and non-responders between the LRP and RRP groups was minimized. Third, only a few patients were treated with nerve sparing. Sexual function is related to HRQOL, but only 10% of the patients wanted preservation of sexual function, which prevented a more detailed examination of this issue. Despite these limitations, the results provide important information on HRQOL after LRP.

In this study, urinary function including incontinence was worse at 36 months after LRP compared with RRP, and had not recovered to baseline. The surgical procedure was a significant factor associated with urinary domains in multivariate analysis, indicating that LRP reduces QOL in comparison with RRP. Improvement of surgical methods, such as robotic surgery and total reconstruction of the pelvic floor, continue to contribute to recovery of urinary incontinence. There have been major changes from open to laparoscopic and robot-assisted surgery in this century, but there is clearly still room for improvement of functional outcomes in radical prostatectomy.

## Conclusion

In this study, we examined prospective longitudinal outcomes of HRQOL after LRP compared with RRP. Urinary function and bother at 36 months were worse after LRP than after RRP. Age, salvage radiotherapy and surgical procedure were associated with urinary incontinence after 36 months. These outcomes may be useful for decision-making in treatment for localized prostate cancer.

## Abbreviations

ADT: Androgen deprivation therapy
EPIC: Expanded Prostate Cancer Index Composite
HRQOL: Health-related quality of life
IPSS: International Prostate Symptom Score
LRP: Laparoscopic radical prostatectomy
MCS: Mental component summary
PCS: Physical component summary
RARP: Robot-assisted radical prostatectomy
RRP: Retropubic radical prostatectomy
SF-8: Medical Outcome Study 8-Items Short Form Health Survey
